# Alignment of multiradiation isocenters for megavoltage photon beam

**DOI:** 10.1120/jacmp.v16i6.5733

**Published:** 2015-11-08

**Authors:** Yin Zhang, Kai Ding, Garth Cowan, Erik Tryggestad, Elwood Armour, Ken Kang‐Hsin Wang

**Affiliations:** ^1^ Department of Radiation Oncology and Molecular Radiation Sciences Johns Hopkins University, School of Medicine Baltimore MD USA; ^2^ Elekta Oncology Systems Crawley UK; ^3^ Department of Radiation Oncology Mayo Clinic Rochester MN USA

**Keywords:** radiation isocenter, quality assurance, acceptance, table rotation

## Abstract

The accurate measurement of the linear accelerator (linac) radiation isocenter is critical, especially for stereotactic treatment. Traditional quality assurance (QA) procedure focuses on the measurement of single radiation isocenter, usually of 6 megavoltage (MV) photon beams. Single radiation isocenter is also commonly assumed in treatment planning systems (TPS). Due to different flattening filters and bending magnet and steering parameters, the radiation isocenter of one energy mode can deviate from another if no special effort was devoted. We present the first experience of the multiradiation isocenters alignment on an Elekta linac, as well as its corresponding QA procedure and clinical impact. An 8 mm ball‐bearing (BB) phantom was placed at the 6 MV radiation isocenter using an Elekta isocenter search algorithm, based on portal images. The 3D radiation isocenter shifts of other photon energy modes relative to the 6 MV were determined. Beam profile scanning for different field sizes was used as an independent method to determine the 2D multiradiation isocenters alignment. To quantify the impact of radiation isocenter offset on targeting accuracy, the 10 MV radiation isocenter was manually offset from that for 6 MV by adjusting the bending magnet current. Because our table isocenter was mechanically aligned to the 6 MV radiation isocenter, the deviation of the table isocentric rotation from the "shifted" 10 MV radiation isocenter after bending magnet adjustment was assessed. Winston‐Lutz test was also performed to confirm the overall radiation isocenter positioning accuracy for all photon energies. The portal image method showed the radiation isocenter of the 10 MV flattening filter‐free mode deviated from others before beam parameter adjustment. After the adjustment, the deviation was greatly improved from 0.96 to 0.35 mm relative to the 6 MV radiation isocenter. The same finding was confirmed by the profile‐scanning method. The maximum deviation of the table isocentric rotation from the 10 MV radiation isocenter was observed to linearly increase with the offset between 6 and 10 MV radiation isocenter; 1 mm radiation isocenter offset can translate to almost 2 mm maximum deviation of the table isocentric rotation from the 10 MV radiation isocenter. The alignment of the multiradiation isocenters is particularly important for high‐precision radiotherapy. Our study provides the medical physics community with a quantitative measure of the multiradiation isocenters alignment. A routine QA method should be considered, to examine the radiation isocenters alignment during the linac acceptance.

PACS number: 87.55.Qr, 87.56.bd, 87.56.Fc

## I. INTRODUCTION

The accurate measurement of a linear accelerator (linac) radiation isocenter is critical and will ultimately impact the quality of radiation therapy, especially high‐precision techniques, such as stereotactic radiosurgery (SRS) and stereotactic body radiation therapy (SBRT). The mechanical isocenter of a linac is defined as a virtual point at which the rotation axes of gantry, collimator, and treatment table intersect in an ideal condition, and is generally assumed to be within a virtual sphere due to mechanical limitations. American Association of Physicist in Medicine (AAPM) Task Group (TG) 40^(1)^ provides quality assurance (QA) guidelines for radiation oncology and one important recommendation is that the coincidence of radiation and mechanical isocenter should be within 2 mm diameter. Later in AAPM TG 142,^(2)^ it is recommended that the coincidence of radiation and mechanical isocenter should be within ±1 mm from baseline for SRS/SBRT. However, neither report provides guidance of quantifying the congruence of the radiation isocenters for different energy modes.

The radiation isocenter of each energy mode can be within 1 mm from mechanical isocenter, complying the TG‐142 recommendation, but the relative distance among radiation isocenters can still be uncertain. Because of different flattening filters and bending magnet and steering parameters, the radiation isocenter of one energy mode can deviate from another if no special effort was devoted during the linac acceptance/commissioning phase. Currently, there is no established method to determine the relative radiation isocenter distance, thus major vendors do not provide the multiradiation isocenters alignment test in their routine acceptance procedure. For high‐precision radiotherapy, such as SRS and SBRT, the multiradiation isocenters alignment is also not included in the related AAPM TG reports.^(3)^ However, the mechanical isocenter and lasers can only be aligned to one radiation isocenter and single radiation isocenter is commonly assumed in treatment planning systems (TPS), so it is critical to align the multiradiation isocenters during acceptance and to establish a routine QA procedure to assure the accurate alignment of the radiation isocenters.

The purpose of this study is to address the importance of the multiradiation isocenters alignment and present an electronic portal imaging device (EPID)‐based imaging procedure to determine the alignment with submillimeter accuracy. Based on the published literatures, this is the first study reporting the concept of the multiradiation isocenters alignment and its corresponding QA procedures. The three‐dimensional (3D) relative distances between the radiation isocenters were measured with an 8 mm ball‐bearing (BB) phantom and the measurements were confirmed using in‐water profile scanning. Per standard practice, our linac mechanical components, such as the table isocenter, are aligned to the 6 MV radiation isocenter. Therefore, to investigate how the misalignment of radiation isocenters could impact clinical practice, we shifted the radiation isocenter of the 10 MV beam away from that of 6 MV by adjusting beam steering parameters. We then investigated the deviation between the table isocentric rotation with that of the 10 MV radiation isocenter. Finally, an EPID‐based Winston‐Lutz test was performed to investigate the radiation isocenter position at various gantry, collimator, and table angles for different energy modes.

## II. MATERIALS AND METHODS

An Elekta VersaHD (Elekta Oncology Systems, Crawley, West Sussex, UK) linac, equipped with the Agility 160‐leaf multileaf collimator (MLC), was used for this study. The available photon energies of the VersaHD are 6 MV, 10 MV, 15 MV, 6 MV flattening filter‐free (FFF), and 10 MV FFF.

### A. Multiradiation isocenters alignment with Elekta BB phantom

**Figure 1:**
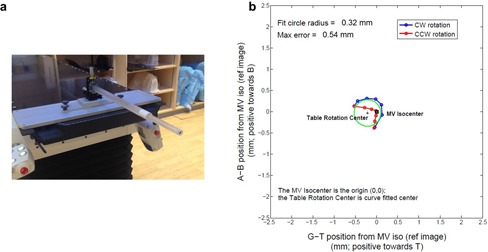
Elekta 8 mm ball‐bearing (BB) phantom (a) mounted to the treatment couch. An example of table rotation analysis (b) with the BB phantom using in‐house MATLAB script. CW = clockwise; CCW = counter‐clockwise. The fit circles for the CW and CCW trajectories are shown. A‐B indicates the A‐side and B‐side, respectively, corresponding to the patient right and left side at supine orientation. G‐T stands for the gun and target direction, respectively. The maximum deviation of the BB phantom during rotation to the radiation isocenter was also derived, indicated as max error in the figure.

An 8 mm BB phantom provided by Elekta has been routinely used for cone‐beam computed tomography (CBCT) kilovoltage (kV) and MV isocenter alignment in our center.^4^, ^5^ During the linac acceptance, we adopted this method for multiradiation isocenters alignment. Briefly, the BB phantom was extended off the treatment table and aligned to the room lasers (Fig. 1(a)), where the nominal radiation isocenter of 6 MV is. Eight EPID images with field size $10\times 10\;{\text{cm}}^{2}$ were acquired at four cardinal gantry angles (0°, 90°, 180°, and 270°) and two collimator angles (90° and 270°). The process follows such that the BB location which minimizes isocentric discrepancies associated with gantry flex, mechanical imperfection over the entire gantry rotation range, and potential diaphragm miscalibration is iteratively determined using the built‐in utility provided by the X‐Ray Volume Imaging (XVI) application (Elekta Oncology Systems). This algorithm was able to localize the center of the steel BB and identify the center of radiation field from the edges of the imaging field by examining the change of pixel value and the predetermined region of interest, and, therefore, the relative 3D shift between the centroid of the BB and radiation isocenter can be derived, per iteration. The algorithm does not depend on the imaging panel calibration. Based on the review of the monthly QA data, the standard deviation of the BB method to locate the radiation isocenter is less than 0.01, 0.02, and 0.04 mm at A‐B axis (patient left and right), Gun‐Target (G‐T), and up‐down axes, respectively. The BB phantom is connected to a three‐axes translational stage, which is able to move the BB in lateral, longitudinal, and vertical direction within 0.01 mm accuracy. The 8 mm BB can therefore be shifted to the radiation isocenter with high precision, and the process is repeated until the desired accuracy of BB isocentricity has been achieved.

To compute the 3D relative radiation isocenter shift, the BB phantom was first placed at the 6 MV radiation isocenter, as described above. With the BB phantom position unperturbed, the portal images of the BB phantom for the other four photon energies were acquired and the shifts of the BB (placed at the 6 MV isocenter) to the respective 3D radiation isocenter locations associated with these energies were also derived. In this way, the relative 3D displacements between the radiation isocenters of the four photon energies to that of 6 MV are known.

### B. Radiation isocenter verification with profile scanning

In addition to the EPID‐based method, a two‐dimensional (2D) profile scan was performed to verify the relative radiation isocenter alignment. The Blue Phantom^2^ water tank (IBA Dosimetry GmbH, Schwarzenbruck, Germany) with OmniPro Accept software (v. 7.4) was used for beam profile scanning. For field sizes $\geq 3\times 3\;{\text{cm}}^{2}$, IBA ion chamber CC04 and PFD photon diode were used, whereas for field sizes $<3\times 3\;{\text{cm}}^{2}$, Sun Nuclear EDGE detector (Sun Nuclear Corporation, Melbourne, FL) was chosen. The scanning detector was placed at the central axis (CAX) of 6 MV photon beam with the gantry set to 0° using CAX searching function provided in OmniPro Accept software. The source‐to‐surface distance (SSD) was set as 100 cm and the 6 MV in‐line and crossline profiles at two depths 2.5 and 12.5 cm were acquired and compared to obtain the CAX position. After the detector was placed at the CAX, the profiles of all five photon energies at depth 10 cm were acquired. The 2D profile shift of other photon energies relative to that of 6 MV was computed for different field sizes in both in‐line and crossline directions.

### C. Table isocenter relative to multiradiation isocenters

The table isocenter should be within ± 1 mm from baseline following Task Group 142.^(2)^ Practically, table isocenter can only be aligned to one radiation isocenter, typically to that of 6 MV, and therefore, the deviation of the radiation isocenters at other energy modes will inevitably impact treatment accuracy. We first aligned the table isocenter to the 6 MV radiation isocenter, based on the Elekta factory table adjustment protocol later modified by Johns Hopkins physics group. We then investigated the deviation between the table isocenter position and the radiation isocenters for all the photon beams. To assess how the radiation isocenter offset relates to the table isocenter, we intentionally shifted the 10 MV radiation isocenter by adjusting the bending magnet parameter, and measured the maximum distance between the radiation isocenter and table isocenter position over 180° table rotation range in both clockwise and counterclockwise directions.

For each photon beam, the BB phantom was first placed with high accuracy to the radiation isocenter, using the above‐described EPID‐based method. One reference EPID image was acquired at table 0° rotation angle, and the MV isocenter can be identified by the BB center. Fourteen subsequent EPID images with the corresponding photon energy were taken at table angles: $-90^{\circ }$, $-60^{\circ }$, $-30^{\circ }$, 0°, 30°, 60°, and 90° at both clockwise and counterclockwise directions. The deviation of the table isocenter from the MV isocenter can be measured by identifying the BB center at these images acquired at the different table rotation angles. An in‐house MATLAB (The MathWorks, Inc., Natick, MA) script^(6)^ was developed to identify the table isocenter by fitting the trajectory of the BB centroid, along the table rotation, with a circular shape. A representative result of the table rotation analysis relative to the 6 MV radiation isocenter is shown in Fig. 1(b). The maximum deviation of the BB phantom during rotation to the radiation isocenter was also derived, corresponding to the maximum possible deviation of the treatment target relative to the radiation isocenter (Fig. 1(b)). The table rotation test was performed for all the five photon energies.

### D. Winston‐Lutz test

After tuning the bending parameters of 10 MV FFF, an EPID‐based variant of a Winston‐Lutz test was performed to evaluate the overall radiation isocenter positioning accuracy at various gantry, collimator, and table angles for all photon energies. The BB phantom was first placed at radiation isocenter, using the described method for the 6 MV beam, and subsequently 20 EPID images, with $4\times 4\;{\text{cm}}^{2}$ field, were acquired at the combination of different gantry, collimator, and table angles: G0C0T0, G0C90T0, G0C180T0, G0C270T0, G90C0T0, G90C180T0, G180C0T0, G180C180T0, G270C0T0, G270C180T0, G225C0T0, G225C180T0, G225C0T90, G225C180T90, G340C0T45, G340C180T45, G20C0T315, G20C180T315, G135C0T270, and G135C180T270, where G denotes gantry, C, collimator, and T, table. The difference between the BB centroid and the center of the image field, which revealed the isocenter shift, was determined on each portal image, and the maximum and mean deviations between the BB centroid and the center of the image field were reported.

## III. RESULTS

### A. Radiation isocenter alignment

Table 1 summarizes the results of radiation isocenter alignment using EPID‐based method and Table 2 summarizes results for the in‐water profile scanning method before tuning the 10 MV FFF beam. The radiation isocenters for 10 MV, 15 MV, and 6 MV FFF were generally consistent, having relative deviations from the 6 MV isocenter of 0.11, 0.28, and 0.19 mm, respectively. However, the radiation isocenter of 10 MV FFF deviated by 0.96 mm from the 6 MV radiation isocenter, primarily in G‐T direction (Table 1).

**Figure 2:**
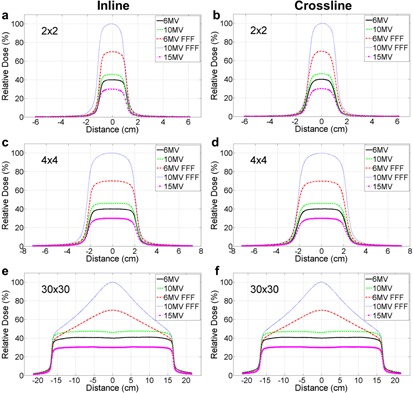
Profile scanning of five photon energies of field size $2\times 2\;{\text{cm}}^{2}$ before tuning the 10 MV FFF beam: (a) in‐line, (b) crossline; of filed size $4\times 4\;{\text{cm}}^{2}$: (c) inline, (d) crossline; and of field size $30\times 30\;{\text{cm}}^{2}$: (e) inline, (f) crossline. The small field sizes were used to confirm the relative shift between 6 MV and other four energies, whereas large field size was used to tune the beam symmetry and flatness. The detector was placed at the central axis (CAX) of 6 MV.

We adopted in‐water profile scanning method to confirm that the radiation isocenter of 10 MV FFF was isolated from the other energy modes. The in‐line and crossline profiles of three field sizes, 2×2, 4×4, and $30\times 30\;{\text{cm}}^{2}$, before tuning 10 MV FFF, are shown in Fig. 2. The profile offset of the other energies relative to the 6 MV profile center is clearly shown in the small field size cases (Fig. 2). From the in‐line and crossline profiles of the $2\times 2\;{\text{cm}}^{2}$ (Fig. 2(a)), the 10 MV FFF center was 1.4 mm toward the T direction and 0.8 mm toward the B direction relative to that of 6 MV (Table 2), confirming the largest offset in the G‐T direction measured using the previous BB method. This finding independently confirmed that the radiation isocenter of 10 MV FFF significantly differed from other four radiation isocenters.

**Table 1:** Multiradiation isocenters alignment before adjusting the 10 MV FFF beam steering parameters for 3D shifts between the radiation isocenter of the 6 MV and other four radiation isocenters measured with EPID‐based method. A‐B represents crossline direction, corresponding to the patient right and left side at supine orientation, respectively. G‐T represents the in‐line direction and stands for the gun and target, respectively

	*6 MV*	*10 MV*	*15 MV*	*6 MV FFF*	*10 MV FFF*
Crossline (mm)	0.05 toward A	0.05 toward A	0.01 toward A	0.04 toward A	0.07 toward A
In‐line (mm)	0.03 toward T	0.12 toward T	0.23 toward G	0.16 toward G	0.99 toward T
Up‐Down (mm)	0.01 toward Up	0.07 toward Up	0.10 toward Up	0.03 toward Up	0.08 toward Up
3D Relative Shift to 6 MV (mm)	0.00	0.11	0.28	0.19	0.96

**Table 2:** Multiradiation isocenters alignment before adjusting the 10 MV FFF beam steering parameters for 2D shifts between the 6 MV CAX and other four CAX measured by the profile scanning method. A‐B represents crossline direction, corresponding to the patient right and left side at supine orientation, respectively. G‐T represents the in‐line direction and stands for the gun and target, respectively

*Field Size* ($cm^{2}$)		*6 MV*	*10 MV*	*15 MV*	*6 MV FFF*	*10 MV FFF*
2×2	Crossline (mm)	−0.1	−0.2	0.1	0.2	0.7
	In‐line (mm)	0.4	−0.2	−0.2	0.3	−1.0
	2D Relative Shift to 6 MV (mm)	0.00	0.61	0.63	0.32	1.61
4×4	Crossline (mm)	0.1	0	0.1	−0.1	0.8
	In‐line (mm)	0.1	−0.5	−0.4	−0.1	−1.2
	2D Relative Shift to 6 MV (mm)	0.00	0.61	0.50	0.28	1.48
30×30	Crossline (mm)	−0.1	−0.1	−0.1	−0.3	0.4
	In‐line (mm)	0.2	−0.3	−0.3	0.2	−0.7
	2D Relative Shift to 6 MV (mm)	0.00	0.50	0.50	0.20	1.03

**Figure 3:**
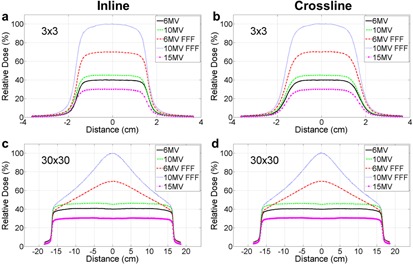
Profile scanning of five photon energies of field sizes $3\times 3\;{\text{cm}}^{2}$ ((a) inline, (b) crossline) and $30\times 30\;{\text{cm}}^{2}$ ((c) inline, (d) crossline) after tuning the 10 MV FFF beam. The detector was placed at the CAX of 6X.

**Figure 4:**
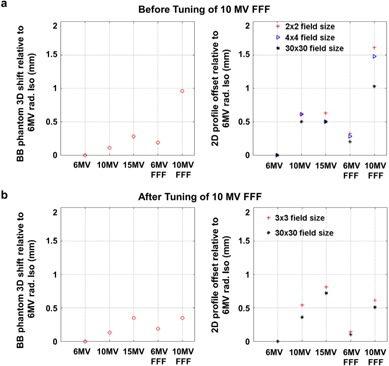
A summary of multiradiation isocenters alignment measured by the EPID‐based method and profile‐scanning method, (a) before and (b) after tuning 10 MV FFF beam parameters.

After our Elekta installation engineer adjusted the beam steering parameters of 10 MV FFF, we repeated the two methods to verify the radiation isocenter alignment during acceptance and commissioning phases, and the measured results are shown in Tables 3 and 4. The relative shifts of 10 MV, 15 MV, and 6 MV FFF to 6 MV measured by the BB phantom method were 0.13 mm, 0.35 mm, and 0.19 mm, respectively, consistent with the measurement prior to the steering parameter adjustment (see Table 1 and 3). Post‐steering, the residual discrepancy between radiation isocenters of 6 MV and 10 MV FFF was significantly reduced to 0.35 mm. The profile scanning method subsequently confirmed this finding, as demonstrated by Fig. 3. The relative 2D shift measured between 6 MV and 10 MV FFF was 0.61 mm for the $3\times 3\;{\text{cm}}^{2}$ field size (Table 4). A summary of multiradiation isocenters alignment measured by the EPID‐based method and profile‐scanning method before and after tuning the 10 MV FFF beam is also shown in Fig. 4. After the beam steering, the radiation isocenter of 10 MV FFF was better aligned with other four energies.

**Table 3:** Multiradiation isocenter alignment after tuning the 10 MV FFF beam steering parameter for 3D shifts between the 6 MV and other four radiation isocenters measured with EPID‐based method

	*6 MV*	*10 MV*	*15 MV*	*6 MV FFF*	*10 MV FFF*
Crossline (mm)	0.06 toward B	0.07 toward B	0.11 toward B	0.03 toward B	0.11 toward B
In‐line (mm)	0.03 toward T	0.07 toward T	0.36 toward T	0.15 toward G	0.35 toward T
Up‐Down (mm)	0.02 toward Up	0.14 toward Up	0.14 toward Up	0.06 toward Up	0.14 toward Up
3D Relative Shift to 6 MV (mm)	0.00	0.13	0.35	0.19	0.35

**Table 4:** Multiradiation isocenter alignment after tuning the 10 MV FFF beam steering parameter for 2D shifts between the 6 MV and other four CAXs measured with the profile scanning method

*Field Size* ($cm^{2}$)		*6 MV*	*10 MV*	*15 MV*	*6 MV FFF*	*10 MV FFF*
3×3	Crossline (mm)	0.5	0.3	0.4	0.6	0.4
	In‐line (mm)	0.1	−0.4	−0.7	0.0	−0.5
	2D Relative Shift to 6 MV (mm)	0.00	0.54	0.81	0.14	0.61
30×30	Crossline (mm)	0.5	0.2	−0.1	0.5	0.4
	In‐line (mm)	0.0	−0.2	−0.4	0.1	−0.5
	2D Relative Shift to 6 MV (mm)	0.00	0.36	0.72	0.10	0.51

### B. Table rotation axis about multiradiation isocenter

During the linac acceptance, the table isocenter was adjusted as close as possible to the 6 MV radiation isocenter. The table isocenter relative the MV isocenters and the maximum distance of the BB phantom during rotation to the radiation isocenters are summarized in Table 5. When the table isocenter was aligned to the radiation isocenter of 6 MV, the initial 10 MV FFF radiation isocenter was 1.13 mm away from the table isocenter resulting in a maximum deviation of the BB relative to the radiation portal of 2.27 mm over the full 180° of table rotation. After tuning the 10 MV FFF photon beam, the table isocenter was 0.63 mm from the 10 MV FFF radiation isocenter revealed by the BB method, resulting in a maximum BB versus radiation portal discrepancy of 0.91 mm, which met our departmental criterion of ≤1 mm.

**Figure 5:**
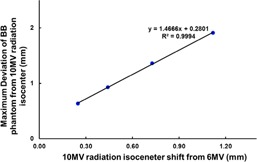
Correlation of the 10 MV radiation isocenter shift from that of the 6 MV and the maximum deviation of the BB phantom ("target") from the 10 MV radiation isocenter due to table rotation. This demonstrates the impact of the misalignment of the radiation isocenters on the accuracy of the target positioning during table rotation.

To further investigate how the radiation isocenter misalignment could impact the accuracy of treatment delivery during table rotation, we manually shifted the radiation isocenter of 10 MV away from that of 6 MV by changing the beam bending parameter. Bending magnet currents at 3.15, 3.25, 3.35, and 3.5 Amp resulted in 0.25, 0.44, 0.73, and 1.12 mm radiation isocenter shifts of 10 MV relative to the 6 MV, respectively. It was observed that the maximum deviation of the BB phantom during table rotation increased linearly with the radiation isocenter shift (Fig. 5; $R^{2}=0.9994$).

**Table 5:** The impact of radiation isocenter misalignment on target positioning accuracy during table rotation

*Energy*	*Table Rotation Isocenter to Radiation Isocenter*	*Maximum Deviation of BB Centroid to Radiation Isocenter During Table Rotation*
6 MV	0.16 mm	0.42 mm
10 MV	0.32 mm	0.63 mm
15 MV	0.30 mm	0.54 mm
6 MV FFF	0.15 mm	0.37 mm
10 MV FFF (Initial)	1.13 mm	2.27 mm
10 MV FFF (Final)	0.63 mm	0.91 mm

### C. Winston‐Lutz test

The Winston‐Lutz test after tuning the 10 MV FFF beam showed that the maximum deviations of the radiation isocenter at different gantry, collimator, and table combinations were 1.16 mm, 1.39 mm, 1.06 mm, 1.36 mm, and 1.03 mm for 6 MV, 10 MV, 15 MV, 6 MV FFF, and 10 MV FFF, respectively (Table 6), complying with our in‐house QA criteria of 1.5 mm maximum deviation. And the mean deviations were 0.57 mm, 0.72 mm, 0.58 mm, 0.66 mm, and 0.63 mm for 6 MV, 10MV, 15 MV, 6 MV FFF, and 10 MV FFF, respectively.

**Table 6:** Winston‐Lutz test results obtained for different energies with EPID on VersaHD after multiradiation isocenters aligned. The maximum and mean deviations of the field and the center of the BB phantom are shown

*Energy*	*Max (mm)*	*Mean (mm)*
6 MV	1.16	0.57
10 MV	1.39	0.72
15 MV	1.06	0.58
6 MV FFF	1.36	0.66
10 MV FFF	1.03	0.63

## IV. DISCUSSION

It is recommended by TG‐142 that the coincidence of collimator, gantry, and couch axes with the radiation isocenter should be within 1 mm diameter for a stereotactic linac.^(2)^ Considering many mechanical and radiation components of a linac involved, it is a nontrivial task to achieve such high accuracy standard. Each individual component must be performed with accuracy much better than 1 mm. It is of great importance to investigate the alignment of multiradiation isocenters to achieve the TG‐142 recommendation. However, based on the best of our knowledge, there is no established method to examine the multi‐isocenter alignment in either vendor‐provided acceptance or published QA procedures. In this study, we introduced an EPID‐based method to quantitatively measure the relative distances among multiradiation isocenters within submillimeter accuracy. We were able to identify the deviation of 10 MV FFF radiation isocenter relative to that of 6 MV and subsequently confirmed the finding with an independent in‐water profile scanning method. It is worthwhile to mention that the water profile scanning was only used for the validation purpose because of its two‐dimensional limitation. Our results also showed that the radiation center misalignment is visually easier to identify using the small field size scanning than the large field size. After Elekta engineers made their best effort adjusting the beam steering parameters, the 10 MV FFF radiation isocenter was aligned to other four energies, and the distance relative to the 6 MV radiation isocenter was 0.35 mm as measured by the EPID‐based method (Fig. 4). Winston‐Lutz test was also performed to assess the overall isocenter positioning accuracy for all the photon energy modes.

By intentionally shifting the radiation isocenter of 10 MV, we were able to assess how the misalignment of radiation isocenters could translate into maximum possible deviation of the radiation isocenter to the "target", simply due to the table rotation. We also observed that the maximum deviation correlated linearly with the radiation isocenter misalignment and 1 mm misalignment can result in maximum of almost 2 mm positioning error during table rotation. This finding is particularly important for SRS/SBRT treatment delivery. One study showed that a 2 mm patient positioning error of a spinal SBRT treatment can result in >5% tumor coverage loss and >25% maximal dose increase to the normal tissues.^(7)^ It is also worthwhile mentioning that, for commissioning the TPS, single radiation isocenter is generally assumed and the profiles of all the energy modes are assumed to be centered.^8^, ^9^ The TPS will not take into account of radiation isocenter offset, and any radiation isocenter misalignment could systematically result in inaccurate treatment plans. One may consider expanding treatment margin to accommodate this systematic offset, which can be clinically significant for stereotactic treatment.

Several methods to measure the radiation isocenter have been developed, such as star‐shot, Winston‐Lutz tests, and EPID‐based method.^10^, ^11^, ^12^ For star‐shots test, films are used to capture the trajectory of radiation fields at different gantry or collimator position, and the central beam lines are identified on the film and used to find the radiation isocenter. Although it is a standard QA test, this method is limited by its two‐dimensional nature. For example, this test is not able to provide radiation isocenter deviation in G‐T direction during gantry rotation, where the largest deviation can possibly occur (Tables 1 and 2). The Winston‐Lutz test was first introduced for SRS in 1988^(10)^ and a circular collimator was used to image a steel ball placed at isocenter with several gantry and table angle combinations. This test has been incorporated into routine QA procedure for isocenter verification.^(13)^ Technically speaking, the Winston‐Lutz test is capable of capturing the multiradiation isocenter misalignment; however, errors associated with misalignments of the gantry, collimator, and table axes are convolved. Therefore, it is not an easy task to identify the origin of a potential QA failure and thereby design appropriate corrective actions. We expect the ultimate test for targeting accuracy remains the Winston‐Lutz test. The method of radiation isocenter alignment proposed in this work can serve as a diagnostic method to evaluate the multi‐isocenter alignment which can affect the outcome of the Winston‐Lutz test (Table 6). The profile scanning method introduced in this study was primarily chosen to be an independent measurement to confirm the misalignment of 10 MV FFF radiation isocenter. Due to the nature of the water scanning configuration, it can only provide the information in 2D. Moreover, the detector was not placed at the isocenter. The offset calculated by this scanning method can be affected by the source to the detector distance. Recently, the EPID‐based method has been used to localize the radiation isocenter by several centers and vendors^14^, ^15^ because of its convenience and accuracy. Therefore, we implemented the EPID‐based method into our routine QA procedures to examine the multiradiation isocenter alignment.

Since there is no flattening filter for FFF beams, to achieve the desired beam profiles the electrons beam needs to be incident upon the thick tungsten target essentially perfectly, without any tilting. Particular attention may be required to monitor the FFF beam profiles during acceptance, and extra effort should be devoted to the alignment of multiradiation isocenters. The methods presented in this study can be adopted into acceptance procedure to examine the multiradiation isocenters alignment.

## V. CONCLUSIONS

In this study, we first investigated the multiradiation isocenters alignment of megavoltage photon beams and developed a procedure to accurately measure the relative displacements of multiradiation isocenters using on‐board EPID. The alignment of the multiradiation isocenters is particularly important for high‐precision radiotherapy, such as SRS and SBRT. A QA method should be established to examine the multiradiation isocenters alignment during the linac acceptance.

## ACKNOWLEDGMENTS

We gratefully acknowledge the support from Elekta, in particular to Kevin Brown, Neil McCann, and Roger Vernon. We also would like to thank Drs. Eric C. Ford and Yidong Yang first developing the in‐house MATLAB QA code.
